# Broad and potent cross clade neutralizing antibodies with multiple specificities in the plasma of HIV-1 subtype C infected individuals

**DOI:** 10.1038/srep46557

**Published:** 2017-04-24

**Authors:** Narayanaiah Cheedarla, K. Lucia Precilla, Hemalatha Babu, K. K. Vidya Vijayan, Manickam Ashokkumar, Padmapriyadarsini Chandrasekaran, Nandagopal Kailasam, Jagadish Chandrabose Sundaramurthi, Soumya Swaminathan, Viswanath Buddolla, S. Kalyanaraman Vaniambadi, V. D. Ramanathan, Luke Elizabeth Hanna

**Affiliations:** 1 HIV/AIDS Division, Department of Clinical Research, National Institute for Research in Tuberculosis, Clinical Research, Chennai, 600031, India; 2 ART Center, Kilpauk Medical College and Hospital, Chennai, 600010, India; 3 Department of Bionanotechnology, Gachon University, San 65, Bokjeong-Dong, Sujeong-Gu, Seongnam-Si, Gyeonggi- Do, 461701, Republic of Korea; 4 Advanced Bioscience Laboratories Inc., Rockville, MD, 20850, USA

## Abstract

Broadly Cross clade Neutralizing (BCN) antibodies are recognized as potential therapeutic tools and leads for the design of a vaccine that can protect human beings against various clades of Human Immunodeficiency Virus (HIV). In the present study, we screened plasma of 88 HIV-1 infected ART naïve individuals for their neutralization potential using a standard panel of 18 pseudoviruses belonging to different subtypes and different levels of neutralization. We identified 12 samples with good breadth of neutralization (neutralized >90% of the viruses). Four of these samples neutralized even the difficult-to-neutralize tier-3 pseudoviruses with great potency (GMT > 600). Analysis of neutralization specificities indicated that four samples had antibodies with multiple epitope binding specificities, *viz*. CD4-binding site (CD4BS), glycans in the V1/V2 and V3 regions and membrane proximal external region (MPER). Our findings indicate the strong possibility of identifying highly potent bNAbs with known or novel specificities from HIV-1 subtype C infected individuals from India that can be exploited as therapeutic tools or lead molecules for the identification of potential epitopes for design of a protective HIV-1 vaccine.

An important goal of Human Immunodeficiency Virus (HIV) research is the development of a vaccine that can elicit highly potent broadly neutralizing antibodies (bNAbs) such as those seen in some of the HIV-infected individuals who are able to partly control HIV infection^1^,^2^. During the acute stage of HIV infection, most individuals develop non-neutralizing antibodies (n-NAbs) that bind primarily to non-functional envelopes and may mediate antiviral activity through Antibody Dependent Cellular Cytotoxicity (ADCC) or Antibody mediated Cellular Phagocytosis (ADCP). About 10–30% of chronically infected individuals are reported to produce bNAbs over a period of time, that are capable of blocking HIV infection through neutralization^3^,^4^,^5^. The sole target for bNAbs is the envelope (Env) on the surface of HIV that helps the virus to infect the host cell. Broadly neutralizing antibodies are categorized into five types based on their target sites on the HIV envelope, viz. CD4 binding site (HJ16, NIH45-46, VRC01-03, VRC06, 3BNC117 etc.), N160 glycan in the V2 apex (PG9, PG16, CAP256-VRC26), N332 glycan at the base of the V3 loop (PGT121, PGT128), gp120-gp41 interface (8ANC195, PGT151 and 35022) and the membrane proximal external region (MPER) (10E8)^6^. High genetic diversity and presence of glycan shield in the HIV Env constitute major hurdles for the development and function of bNAbs^7^.

Several research groups have identified and characterized a number of bNAbs with great breadth and potency employing advanced immunological techniques^8^,^9^,^10^,^11^. In the recent years, a number of bNAbs capable of strongly neutralizing HIV-1 clade C strains have also been identified^12^. HIV-1 subtype C is the predominant strain in the HIV epidemic and is responsible for >50% of infections worldwide and >90% of infections in India (UNAIDS 2016 and NACO 2016), and therefore, identification of more bNAbs that can strongly neutralize HIV-1 subtype C viruses is a global priority. In the present study, we screened plasma of 88 HIV-1C infected individuals to identify broadly neutralizing antibodies with good breadth and potency and characterized their neutralization specificities.

## Results

### Clinical profile of study subjects

The study population comprised of 101 HIV-1 infected ART naïve individuals (41 males, 59 females, 1 transgender), aged between 22 and 53 years. Most individuals (n = 65) had CD4+ T cell counts >350 cells/mm^3^ (median 500 cells/mm^3^). CD4+ T cell count could not be performed on 4 samples due to sample lysis. Viral load was estimated using the COBAS Amplicor HIV-1 Monitor v1.5 and found to range between 400–750,000 copies/ml (median 26,212 copies/ml). Table 1 provides the demographic details and clinical profile of the study participants; complete clinical and immunological data are provided as a Supporting Table (Table S1).

### Neutralization of plasma samples

Thirteen samples were excluded from testing either due to insufficient sample volume or due to very recent infection. The remaining 88 plasma samples were screened for HIV-1 neutralization activity against a panel of 6 tier-1 pseudoviruses listed in Supplementary Table 2 (Table S2). Fifty eight samples were found to neutralize 4 or more of the viruses, and eight neutralized all the 6 pseudoviruses. >60% neutralization was considered as good breadth of neutralization as defined by Montefiori (2005). The 58 samples were further tested against a panel of six tier-2 pseudoviruses. Thirty plasma samples were found to neutralize 4 or more pseudoviruses, and six of them neutralized all the 6 pseudoviruses. The 30 samples were screened with a panel of six tier-3 pseudoviruses. Twelve samples , namely NAB001, NAB004, NAB016, NAB033, NAB046, NAB059, NAB062, NAB063, NAB065, NAB069, NAB120 and NAB122 neutralized 4 or more pseudoviruses; hence they were considered as broadly cross-clade neutralizing (BCN) samples. The results of the neutralization assay are provided in Table S2 and the heat maps of neutralization activity against the 3 panels of the pseudoviruses are provided as Supplementary Figs S1, S2 and S3.

### Potency of neutralization of BCN samples

**Figure 1:**
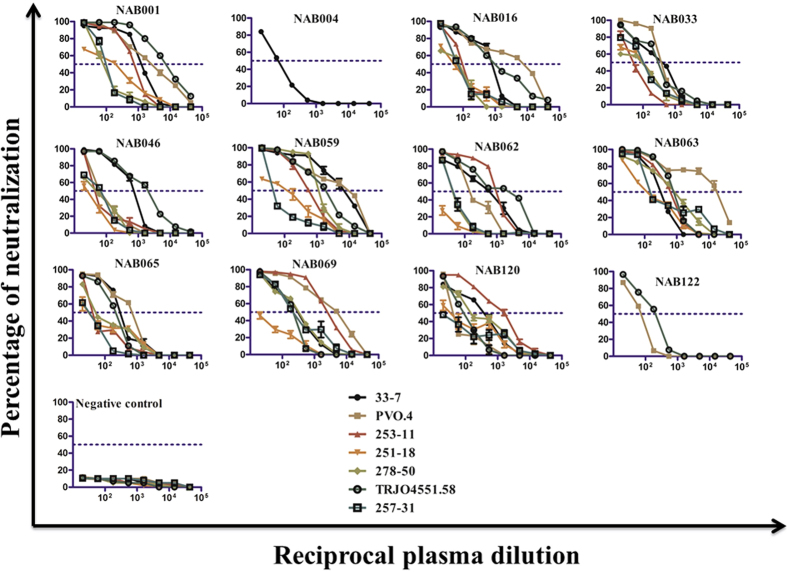
Neutralization potency of 12 BCN plasma samples. Neutralization titration (1:20 to 1:43740) analysis was performed against 7 tier-3 pseudoviruses in TZM-bl cells. Most of the BCN plasma were able to neutralize tier-3 pseudoviruses which are highly resistant to neutralization with high efficiency as seen by the high ID_50_ values.

The neutralization titration assay was performed for 10 of the 12 BCN samples with a panel of 7 tier 3 pseudoviruses listed in Table 2 (two samples were titrated with one or two pseudoviruses only due to insufficient sample volume). The ID_50_ was calculated through a dose-response curve fit with non- linear function using GraphPad prism5.0 (Fig. 1). Geometric mean titer (GMT) was calculated for each plasma against all the tier-3 pseudoviruses (Table 2). Four plasma samples (NAB001, NAB059, NAB063 and NAB069) neutralized all the tier-3 pseudoviruses with GMT >400 and had ID_50_ values >50. The GMT of the remaining samples (NAB016, NAB033, NAB046, NAB062, NAB065 and NAB120) ranged between 100 and 400. Thus, most of the BCN samples tested exhibited high potency for neutralization.

### Antibodies to linear peptides of 93IN101gp160

**Figure 2:**
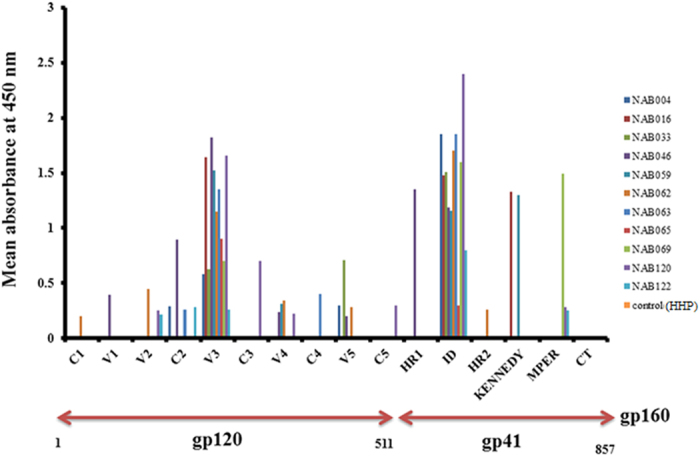
Linear PepScan of 93IN101 gp160. Total of 192 peptides, 15 amino acid long with 11 amino acid overlapping sequence covering the entire 93IN101 envelope (gp160) protein were used. Plasma samples (n = 11) from BCN samples were tested at a dilution of 1:50 for the identification of binding reactivity. Healthy Human Plasma (HHP) was used as the negative control. Mean absorbance was calculated from each experiment performed in duplicate and on two independent occasions.

The BCN samples were tested in an ELISA with linear peptides spanning the full length envelope protein of the Indian HIV-1 subtype C clone 93IN101, in order to identify the binding specificities of the antibodies in the plasma. Eleven BCN samples were used for this analysis (one sample could not be tested due to insufficient volume). All 11 samples had antibodies that bound to the V3 loop and Immunodominant (ID) region of gp41. Three samples had antibodies that bound to the MPER peptides (Fig. 2). Some of the BCN samples exhibited binding activity to discontinuous epitopes spread across the HIV Env Gp160. None of the samples had antibodies against the Cytoplasmic tail (CT) of the HIV-1 envelope.

### CD4 binding site (CD4BS) antibodies

**Figure 3:**
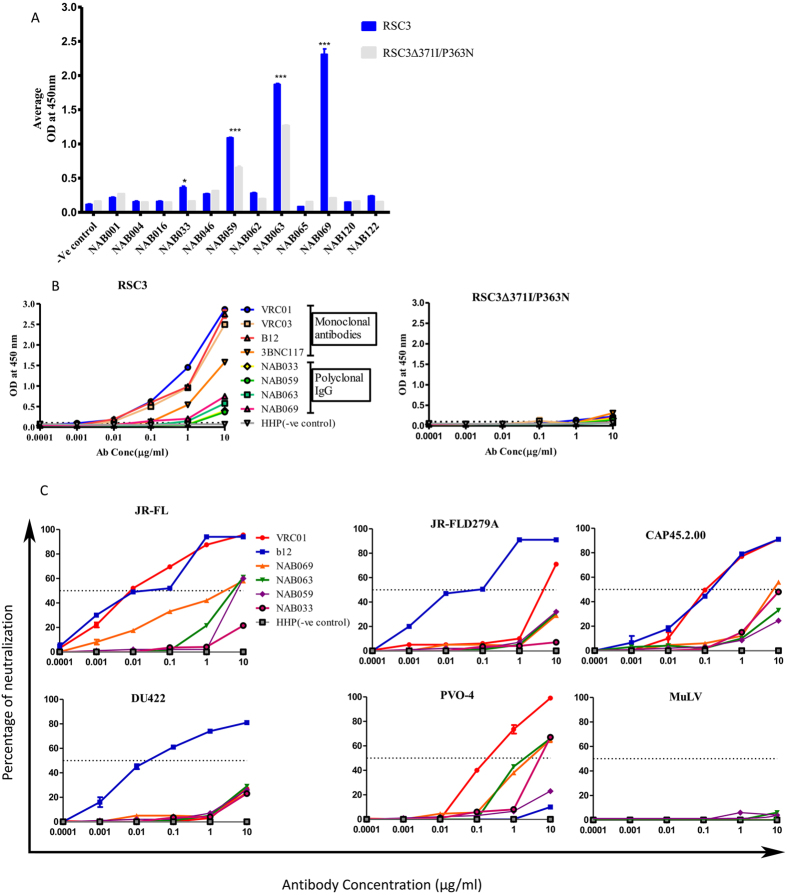
Identification of CD4-binding site antibodies. (**A**) ELISA with RSC3 and RSC3∆371I/P363N recombinant proteins. 12 BCN plasma samples were tested at a dilution of 1:100 to screen for the presence of CD4 binding site directed antibodies. Four of the 12 BCN plasma samples tested demonstrated significantly stronger binding to the wild type RSC3 protein as compared to the mutant protein. (**B**) RSC3 and RSC3∆371I/P363N specific antibodies were eluted from four BCN plasma samples which revealed the presence of CD4 binding site targeting antibodies (NAB033, NAB059, NAB063 and NAB069) using tosyl activated MyOne Dynabeads. ELISA was again performed with eluted IgG antibody (concentration 10 to 0.0001 μg/ml) with RSC3 and RSC3∆371I/P363N recombinant proteins at 2 μg/ml. Monoclonal antibodies VRC01, VRC03, B12 and 3BNC117 were used as positive controls and HHP as negative control. (**C**) Neutralization titer (IC_50_) assay performed with eluted IgG antibody (concentration 10 to 0.0001 μg/ml) against four pseudoviruses and one mutant (JR-FL D279A). VRC01 and b12 MAbs were used as positive control bNabs against CD4BS and MuLV, as negative control.

The 12 BCN plasma samples were screened for the presence of CD4BS antibodies using the recombinant CD4BS protein RSC3 and its mutant form RSC3∆371I/P363N. Four samples (NAB033, NAB059, NAB063 and NAB069) had varying amounts of RSC3 directed CD4BS antibodies (55, 40.2, 32.3 and 91% respectively), as indicated by their ability to bind selectively to the wild type protein but not to the mutant form (i.e., difference in binding activity between RSC3 and RSC3∆371I/P363N was significant, p < 0.05). RSC3 specific IgG was eluted from these four samples and binding activity was compared with that of well characterized MAbs such as VRC01, VRC03, b12 and 3BNC117 (Fig. 3A,B and Table 3). Further, neutralization titer assay was performed for RSC3-specific IgG eluted from the four BCN plasma samples with a VRC01- resistant and sensitive pseudovirus (DU422 and CAP45.2.00), a sensitive and resistant tier-2 pseudovirus (JR-FL wild type and JR-FL mutant D279A), as well as a tier-3 pseudovirus (PVO-4). Known CD4BS monoclonal antibodies VRC01 and b12, were used as positive controls. The known CD4BS antibodies showed stronger neutralization of the wild type (sensitive) viruses than IgG eluted from BCN samples as the former were monoclonal and the latter were polyclonal in nature. Two BCN plasma samples (NAB069 and NAB063) strongly neutralized JR-FL WT (IC_50_ at 1.18 and 1.28 μg/ml respectively) but not the mutant JR-FL D279A, while NAB059 weakly neutralized JR-FL WT (IC_50_ at 9.1 μg/ml). NAB069, NAB063 and NAB033 neutralized tier-3 HIV-1 subtype B pseudovirus PVO-4 at >5 μg/ml. NAB069 alone neutralized CAP45.2.00 virus at 9 μg/ml (Fig. 3C and Table 4).

### Antibodies targeting glycans at positions 160 and 332 on the gp120 trimer

**Figure 4:**
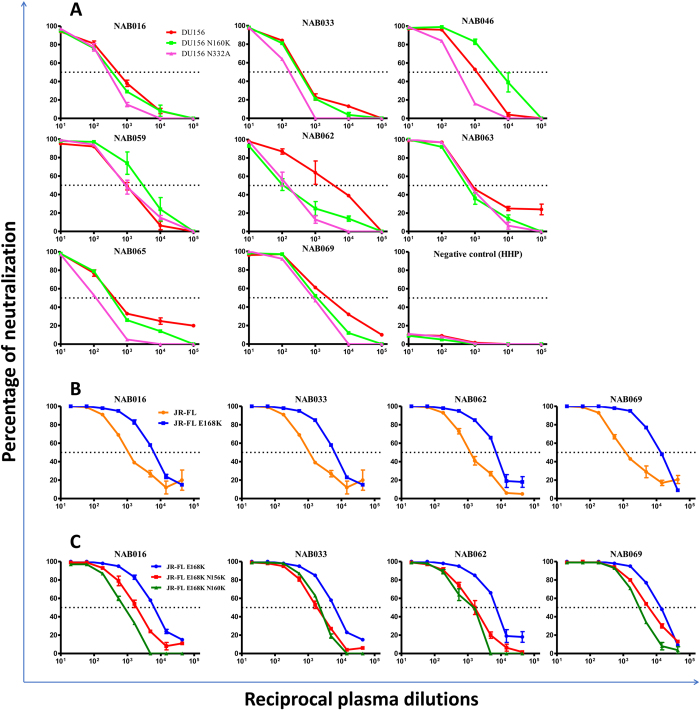
Neutralization sensitivity assay to detect Glycan-dependent antibodies. (**A**) Neutralization curves of BCN plasma samples against HIV-1 subtype C tier-2 pseudoviruses Du156.12 WT and mutants with substitutions at glycan positions 160 and 332. Neutralization titration assay was performed with BCN plasma samples in duplicates at dilutions 1:10 to 1:100000 on two independent occasions. (**B**) Sensitivity analysis of JR-FL wild type (WT) and E168K mutant in the selected BCN plasma samples for the PG9/PG16 recognizing epitopes. (**C**) Neutralization activity against glycan positions at N156 and N160 in the JR-FL E168K mutated strain for the confirmation of PG9/PG16 bNAbs. The assays were performed in duplicates on two independent experiments with dilutions ranging from 1:20 to 1:43740.

Neutralization assay was performed for 8 of the 12 BCN samples, for which sufficient volume of plasma was available, with a wild type tier-2 HIV-1 subtype C virus (DU156), and its mutant forms (DU156N160K and DU156N332A). All 8 samples showed strong neutralization activity against DU156WT (Fig. 4A, Table 5). Four samples showed significantly lower levels of neutralization of DU156N160K; neutralization activity of one sample (NAB062) was 25 fold lower with the DU156N160K mutant as compared to DU156WT, but <3 fold with the other three samples (NAB016, NAB033 and NAB069). The remaining four samples (NAB046, NAB059, NAB063 and NAB065) showed a higher level of neutralization of DU156N160K than the WT virus, indicating that antibodies in these samples do not specifically target N160 glycan but target other regions of the HIV-1 Env.

The four samples (NAB016, NAB033, NAB062 and NAB069) that showed varying levels of dependence on N160 glycan were further studied to understand whether the glycan dependent antibodies in these individuals were similar to those belonging to the PG9/PG16 series by performing neutralization assay. It is known from literature that PG9/PG16 bNAbs do not neutralize the HIV-1 subtype B JR-FL WT strain due to presence of glutamic acid (E) at 168 position. However, when E is replaced with lysine (K) in the WT JR-FL strain by single mutation, it turned sensitive to neutralization by PG9/PG16 antibodies^13^. We therefore used these two pseudoviruses (JR-FL WT and JR-FL E168K) for further analysis of PG9/PG16 like bNAbs. Four samples strongly neutralized (>3 fold) the mutant pseudovirus than the JR-FL WT (Fig. 4B and Table 6). Neutralization was also performed for the identification of positional dependence using two double mutants JR-FL E168K N156K and JR-FL E168K N160K (Fig. 4C). Three of the four samples (NAB016, NAB062 and NAB069) showed reduced neutralization of both double mutants (3–5 fold reduction) as compared to the single mutant (Table 7). One sample (NAB033) showed <3 fold reduction in neutralization of both the double mutants.

Further, we also observed lower levels of neutralization of the mutant virus, DU156N332A, by seven of the eight BCN samples as compared to DU156WT; the decrease was >3 fold in three samples (NAB046, NAB062 and NAB069) and 1.5–2.5 fold in four samples (NAB016, NAB033, NAB059 and NAB065). NAB062 showed the maximum reduction in neutralization of 24.3 fold as compared to the WT DU156 (Table 5).

### MPER specific neutralizing antibodies

To identify MPER-specific neutralizing antibodies, we selected three plasma samples NAB069, NAB120 and NAB122 which showed binding to overlapping MPER peptides in the initial PepScan analysis (Fig. 2). None of the three samples neutralized HIV-2 7312A strain (Table 8). NAB069 showed a 1.5 fold higher titer for neutralization of chimeric virus containing the subtype B MPER (C1) as compared to the one containing the subtype C MPER (C1C), suggesting the presence of 2F5, 4E10 or Z13e1-like neutralizing antibodies. In contrast, for NAB122, there was a significant drop in ID_50_ value with virus containing the subtype C MPER as compared to the one containing the subtype B MPER. However, the two plasma samples (NAB069 and NAB122) neutralized C6 strain which recognizes the 4E10 epitope, WFDIT, which is present in both subtypes B and C. NAB120 did not neutralize any of the chimera viruses.

It is reported that neutralization of MPER chimera viruses need not necessarily result in neutralization of HIV-1 primary isolates, since HIV-2/HIV-1 chimera viruses are more sensitive to the 4E10 and Z13e1 MAbs than HIV-1 primary isolates^14^. Therefore, to evaluate further the neutralization ability of NAB069 and NAB122 plasma, we eluted polyclonal IgG from these samples using pIndie full length MPER peptide coated tosyl-activated Dynabeads. While IgG eluted from the blank beads and HHP samples did not show neutralization activity against any of the tested pseudoviruses (Supporting Table S3 and Fig. S4), polyclonal IgG eluted from NAB122 exhibited moderate neutralization breadth and potency against all the tested tier-2 cross-clade pseudovirus panel (Table 9). NAB069 neutralized all viruses except RHPA pseudovirus (subtype B), indicating the presence of MPER specific bNAbs in these two samples.

## Discussion

In the recent years, enormous amount of research has been focused on the identification of broadly neutralizing antibodies from many parts of the world^15^,^16^,^17^,^18^ and several broad and potent neutralizing antibodies have been identified from HIV-infected individuals^19^,^20^,^21^,^22^,^23^. However, Indian research in this line have been limited to a few groups^24^,^25^,^26^,^27^. Given the preponderance in the global epidemic of the subtype C virus which is also the predominant circulating subtype in the Indian population, more studies in HIV-1 subtype C infected individuals would possibly lead to the identification of more potent bNAbs and novel epitopes that can be exploited for HIV vaccine research.

In the present study, plasma of 88 asymptomatic, ART naïve HIV-1 subtype C infected individuals from a South Indian HIV-1 cohort were screened for the presence of bNAbs. Twelve of the 88 samples (14%) strongly neutralized >90% of the tier 1, 2 and 3 viruses tested and have been categorized in this study as broadly cross-clade neutralizing (BCN) samples. Previous studies have also reported that about 10-30% of HIV-1 infected individuals develop bNAbs sometime during the course of infection^28^,^29^. Interestingly, we observed that all the BCN samples identified in our study neutralized subtype C pseudoviruses very strongly, endorsing recent reports on strain specific neutralization potential of NAbs^30^,^31^.

The BCN samples identified in the present study were characterized for their potency by determining their ID_50_ titers against seven difficult-to-neutralize tier-3 pseudoviruses. Four samples (NAB001, NAB059, NAB063 and NAB069) demonstrated high potency of neutralization, with GMT >600; one sample (NAB059) had a GMT >1100. The remaining six samples exhibited moderate potency, with GMT ranging from 100 to 400. Our findings are in agreement with previous reports that approximately 1% of the HIV-1 infected individuals may develop antibodies with strong potency and great breadth of neutralization^28^.

PepScan analysis showed that most of the samples had antibodies that bound strongly to the V3 and ID regions of gp160. Similar observations were also made by other groups of investigators^24^,^32^ who reported that most of the HIV-1 infected individuals harbour such antibodies, but these are generally non-neutralizing in nature. Two samples (NAB016 and NAB059) showed reactivity with the Kennedy epitope (KE; PRGPDRPEGIEEEGGERDRDRS)^33^ present in the transmembrane (TM) region of gp41 (amino acids 731 to 752 as on HXB2)^34^. This region is known to contain three epitopes PDRPEG (KE1), IEEE (KE2) and ERDRD (KE3), and antibodies to these epitopes are thought to bind to HIV-1 particles after they attach themselves to the cell receptors^35^. In general, the antibody response to the Kennedy epitopes in HIV-1 infected individuals is thought to be very weak^36^. Interestingly, the Indian reference strain, 93IN101 (pIndie), has amino acid substitutions in KE1 and KE3, resulting in an altered amino acid composition of the epitopes (PDRLGR and EQDKDR respectively). NAB016 and NAB059 exhibited a high binding affinity to PDRLGR, suggesting that changes in KE1 of subtype C has relevance to improved binding antibody response, which might or might not translate to inactivation of HIV-1 by neutralization. MPER comprises the last 23 residues of the gp41 ectodomain. Several well characterized NAbs like 2F5, Z13e1, 4E10 and 10E8 bind to linear epitopes in MPER^17^,^37^. In our study, three samples (NAB069, NAB120 and NAB122) showed binding reactivity to MPER peptides.

The CD4 receptor on the target cell plays a major role in viral entry by interacting with the CD4 binding site present on the HIV envelope. Before 2009, b12 was the only CD4BS bNAb that was identified. However, since 2009, several CD4BS bNAbs with greater breadth and potency of neutralization than b12 have been isolated from HIV-1 infected individuals, such as VRC01-03, VRC23, NIH45-46, 3BNC117, etc^38^, and two of these, VRC01 and 3BNC117, are currently being tested for use as therapeutic vaccines against HIV-1^39^,^40^. We first tested plasma binding activity to the wild type RSC3 protein which selectively binds to VRC01-like CD4BS specific bNAbs, and a mutant form of the protein which shows decreased binding to CD4BS bNAbs^25^,^41^. We identified four samples containing VRC01-like CD4BS antibodies, as demonstrated by their ability to preferentially bind to the RSC3 WT protein but not to the mutant form. In order to determine the neutralizing potential of the CD4BS specific antibodies in these samples, we eluted IgG from the plasma and tested them in a neutralization assay with tier-2 and tier-3 pseudoviruses. Two of the four samples, NAB069 and NAB063, exhibited strong neutralization of the tier-2 virus JR-FL WT and tier-3 HIV-1 subtype B pseudovirus PVO-4 but diminished neutralization of JR-FL D279A, just like VRC01. These observations provide clear evidence to show that these samples contain CD4BS specific antibodies with neutralization ability. Our findings mirror those of Landais *et al*.^42^ and others that although RSC3 binding activity may be found in some HIV-1 infected individuals^42^, RSC3 reactive antibodies mediating broad neutralization were only detected in very few individuals. A major limitation of the RSC3 binding approach as observed by previous investigators (Walker *et al*.^14^) is that some CD4BS bNAbs may not bind to the RSC3 protein, necessitating the use of additional recombinant gp120 (rgp120) core molecules^14^,^43^. However, we could not perform further experiments with rgp120 adsorbed antibodies due to insufficient sample volume.

It is well known that HIV uses glycan shields to escape attack by the host immune system. The glycan shields hinder accessibility to the neutralizing antibody binding sites. Thus, identification of bNAbs that target some of the critical glycans on the HIV envelope could pave the way for designing an effective vaccine against HIV^44^. Potential N-linked glycans at positions 160 and 332 on the HIV envelope are known to be very crucial for the binding of glycan dependent antibodies belonging to the PG9/PG16 and PGT series, respectively^13^,^45^. We evaluated the BCN samples identified in our study for the presence of potential N-linked glycan dependent antibodies. For this purpose, we performed neutralization assay with a subtype C pseudovirus, DU156WT, and its mutant forms DU156N160K and DU156N332A. Studies have shown that viruses, mutated at position 160 (V2 loop) escape neutralization by PG9/PG16 antibodies^26^,^46^. We identified four samples (NAB016, NAB033, NAB062 and NAB069) that strongly neutralized DU156WT but showed reduced levels of neutralization of the mutant virus DU156N160K, suggesting that these samples are likely to contain PG9/PG16 like antibodies. One of the four samples, NAB062, showed a 25-fold reduction in neutralization of DU156N160K as compared to DU156WT, indicating the strong specificity of the antibodies present in this sample to this epitope. In addition, these four samples also demonstrated strong neutralization of the JR-FL E168K mutant pseudovirus as compared to WT JR-FL pseudovirus, providing further support for the PG9/PG16-like specificity of the antibodies in these samples. This is due to the presence of glutamic acid (E) at 168 position in JR-FL that masks the exposure of asparagine (N) glycans at positions 156 and 160 thereby abrogating the binding of PG9/PG16 like antibodies to this epitope^13^. We found a further reduction in the neutralization activity of the plasma on the double mutants (JR-FL E168K N156K and JR-FL E168K N160K) as compared to the single mutant, providing further proof to document the presence of PG9/PG16 like bNAbs in these samples.

Interestingly, two of the samples (NAB033 and NAB069) that showed PG9/PG16-like neutralization activity also contained CD4BS antibodies as seen in the previous experiment. Four other samples (NAB046, NAB059, NAB063 and NAB065) showed a higher level of neutralization of the mutant virus DU156N160K, than the WT virus indicating the possibility that these samples contain NAbs that are independent of glycan at N160 but target other regions of the HIV-1 Env.

The PGT series of antibodies (PGT 121-like and PGT128-like) bind to the glycan site N332 from various angles and neutralize HIV-1^38^,^47^. To identify if antibodies with similar specificities were present in any of our BCN samples, we analyzed the plasma for neutralization activity against the subtype C virus, DU156WT and mutant DU156 N332A pseudovirus. Seven BCN samples neutralized DU156WT indicating the presence of antibodies belonging to the PGT series. Similar observations of a high frequency of N332 glycan-dependant antibodies among HIV-1 subtype C infected individuals from sub-Saharan Africa was recently reported by Landais *et al*.^42^. Of the seven samples, one sample NAB062, showed 24.3 folds reduction in neutralization of the mutant as compared to the WT. Two other samples, NAB046 and NAB069, also showed a reduction of 3.3 and 3.5 fold in neutralization of the mutant as compared to WT virus. This suggests the possibility that the above three samples could contain antibodies similar to the PGT series of antibodies. To our knowledge, this is the first study from India to demonstrate the presence of PGT series of bNAbs among HIV-1C infected individuals from the country. Of special interest, we noticed that sample NAB069 had antibodies with multiple specificities, such as CD4BS, V2 glycans and V3 glycan.

The three samples (NAB069, NAB120 and NAB122) which showed reactivity to MPER-specific peptides in the PepScan analysis were analyzed for neutralization potential using HIV-2/HIV-1 MPER chimera pseudoviruses. NAB069 was found to strongly neutralize both subtype B and C chimeric viruses, while NAB122 neutralized them weakly. We further evaluated the neutralization ability of IgG eluted from these two samples for their neutralization ability, and found them to show moderate neutralization breadth and potency against majority of the tier-2 cross-clade pseudoviruses confirming the presence of MPER specific bNAbs in these samples. Our results are in agreement with the previous reports that NAbs directed against HIV-2/HIV-1MPER have moderate breadth and potency of neutralization against HIV-1 pseudoviruses^3^,^14^,^28^,^48^. Since the MPER peptide is known to play a major role in the fusion of viral and host cell membranes^49^, our findings encourage further characterization of antibodies from these two samples.

In summary, our study has identified broad and potent neutralizing antibodies in plasma of a subset of HIV-1 infected individuals from India. Four of the BCN samples had neutralizing activity with binding specificity to more than one vulnerable site on the HIV-1 Env (Table 10). One sample, NAB069, was found to have neutralization activity with binding specificity to all the four vulnerable sites on the HIV-1 Env, suggesting that this sample is worthy of further investigation and characterization. A few other investigators have also reported the identification of bNAbs targeting multiple vulnerable sites on the HIV envelope from a single donor^14^,^48^,^50^,^51^,^52^, but to our knowledge, this is the first study from India that has identified second generation broad cross clade neutralizing antibodies belonging to the CD4BS, PG9/PG16, PGT series and MPER in HIV-1C infected individuals. Further work is ongoing to isolate and characterize monoclonal antibodies from a few of these samples.

## Methods

### Ethics Statement

The study was approved by the Institutional Ethics Committee of the National Institute for Research in Tuberculosis (TRC IEC No: 2011001) and all experiments were performed in accordance with relevant guidelines and regulations. Sample collection was done after obtaining written informed consent from the study participants.

### Study population and sample collection

A total of 101 HIV-1–infected subjects attending the ART Centre at the Kilpauk Medical College and Hospital, Chennai, India, during the period April 2011 to September 2012, were recruited for the study. All study subjects were asymptomatic and naïve to ART at the time of sample collection. Fifteen milliliters of venous blood was collected in K_3_-EDTA treated blood collection tubes. Plasma was separated by centrifugation at 300 g and stored as 1 ml aliquots at −80 °C until use. The plasma samples were heat inactivated at 56 °C for 1 hour before use.

### Production and titration of Pseudoviruses

A panel of 18 pseudoviruses belonging to different clades and all 3 tiers were selected from the standard panel of pseudoviruses obtained from NIH AIDS Reagent program, and used to screen plasma of HIV-1-infected individuals for the presence of bNAbs. In addition, single residue mutant pseudoviruses (DU156 N160K, DU156 N332A) and chimeric viruses (7312A, C1, C1C, C6 and C7) were obtained as a kind gift from Dr. Lynn Morris (NICD, Johannesburg) and Dr. Jayantha Battacharya (THSTI, India), JR-FL WT plasmid from Muzafar Jan and Dr. Sunil Arora (PGI-Chandigarh, India), JR-FL E168K and its mutants N156K and N160K as well as MuLV plasmids from Dr. Raghavan Varadharajan (IISc, Bangalore, India). Pseudo viruses belonging to clades A, B, C, A/E and A/G, RSC3 WT and RSC3 Δ371I/P363N recombinant proteins were obtained from the NIH AIDS Reagent Program. Pseudoviruses were prepared by co-tranfection of pEnv and pSG∆Env back bone plasmid into 293T cells using standard Calcium Phosphate method and titrated on TZM-bl cells^53^,^54^. Neutralization potential of the plasma samples was determined based on an observed reduction in luciferase gene expression after a single round of infection in TZM-bl cells with env-pseudotyped viruses as described^53^,^54^. Murine leukemia virus (MuLV) was used as a control virus.

### Peptide – binding antibody ELISA

Fifteen amino acid long linear peptides with 11 amino acid overlap spanning the entire length of gp160 of the Indian subtype C virus 93IN101 were synthesized commercially (Infinity Biotech and Resource Inc., PA). The peptides were adsorbed onto 96-well ELISA immuno maxisorp plates (Thermo Fisher) at a concentration of 5 μg/ml in 100 mM NaHCO_3_, pH 9.6, by overnight incubation at 4 °C and ELISA was performed as described previously^55^ to map the epitope specificity of samples demonstrating good breadth and potency of neutralization. Each experiment was repeated three times. Healthy Human Plasma pool (HHP) was included as the negative control in all experiments. The samples were subsequently tested in an ELISA with recombinant proteins RSC3 and RSC3∆371I/P363N to look for the presence of CD4 binding site (CD4BS) antibodies.

### Protein-paramagnet bead coupling, adsorption and elution of RSC3- specific antibodies

Paramagnetic polystyrene, Tosylactivated MyOne Dynabeads (Life Technologies) were coupled with recombinant HIV-1 RSC3 and double mutant RSC3Δ371I/P363N proteins or pIndie MPER peptide as per the manufacturer’s instructions. Briefly, 0.1 mg of protein or 1 mg of MPER peptide was coupled to 0.50 mg or 5 mg of tosylactivated magnet MyOne Dynabeads respectively. Coupling was done at room temperature (RT) in a total volume of 1.25 ml in coupling buffer (0.1 M sodium borate buffer [pH 9.5] with 1 M ammonium sulfate) with gentle rocking for 48 hours. The Dynabeads and bound protein were separated from the coupling buffer with a magnet and resuspended in 5 ml of blocking buffer (PBS [pH 7.4] containing 0.5% [wt/vol] bovine serum albumin [BSA] and 0.05% Tween 20) for an additional 48 hours. The blocking buffer was removed by aspiration, and the protein-coupled Dynabeads were washed two times with 5 ml of wash buffer (PBS [pH 7.4] containing 0.1% BSA and 0.05% Tween 20). The beads were then resuspended in 0.5 ml of storage buffer (PBS [pH 7.4] supplemented with 0.1% BSA, 0.05% Tween 20 and 0.02% sodium azide + protease inhibitors) and stored at 4 °C. On the following day, the protein-coupled beads were washed three times with Dulbecco modified Eagle medium (DMEM) containing 10% fetal bovine serum (FBS) and incubated in DMEM containing 10% FBS at room temperature for 30 minutes to block nonspecific binding to the beads. Samples exhibiting strong neutralization activity were diluted 1:50 in DMEM containing 10% FBS, and 1 ml of the diluted plasma was incubated with 0.5 ml of beads at room temperature for 30 minutes. This step was repeated three times for the better removal of antibodies. Beads coupled with RSC3 or MPER-specific antibodies were stored in PBS containing 0.2% BSA and 0.02% sodium azide at 4 °C. The remaining plasma was centrifuged three times at 16,100 × g for 5 minutes each time to remove any residual Dynabeads and used subsequently for ELISA and neutralization assays. BSA-coated beads or blank beads were used as negative controls. The protein-coupled beads were washed three times with PBS containing 500 mM NaCl and once with PBS, and the antibodies were eluted by stepwise decrease in pH. First, the beads were mixed with 100 mM glycine-HCl elution buffer (pH 2.7) for 30 seconds. The beads were then centrifuged for 30 seconds and held in place at the bottom of the tube with a magnet. The acid-eluted solution containing IgG was quickly removed and placed in a separate tube containing 0.5 ml of 1 M Tris (pH 9.0) buffer to reach pH 7.0 to 7.4. This process was repeated three times. The eluted IgG was diluted in DMEM and concentrated over a 30-kDa Centricon plus filter (Millipore Corp.). Subsequently, the same procedure was performed on the beads at an elution pH of 2.2 to recover any IgG resistant to elution at pH 2.7. The IgG fractions recovered by both acid elution steps were combined and the concentration of the combined IgG fractions was measured using nanodrop technology (Thermofisher). The IgG was further characterized by ELISA using RSC3 protein and its double mutant RSC3Δ371I/P363N. Known monoclonal neutralizing antibodies (MAbs) VRC01, VRCO3, b12 and 3BNC117 that bind specifically to the CD4BS were used as positive controls.

### Virus Neutralization assay

Neutralization potential of plasma samples was evaluated using the standard neutralization assay^54^. Briefly, TZM-bl cells were infected with the pesudoviruses in the presence or absence of plasma. After 48 hours, 100 μl of BriteLite (PerkinElmer) substrate was added to the wells. 100 μl of supernatant was transferred to a solid opaque plate and luminescence was measured as relative luciferase units (RLU) using the Vector 3 luminometer (Perkin Elmer). Initial neutralization screening assays were performed at a plasma dilution of 1:10 dilution. Subsequently, for the determination of IC_50_/ID_50_ values, serial dilutions of the plasma samples ranging from 1:20 to 1:43,740 or 1:10 to 1:100,000 were used. Anti-glycan and anti-MPER specific neutralization activity was evaluated using Du156WT, Du156N160K, Du156N332A, JR-FLWT, JR-FL E168K, JR-FL E168K N156K, JR-FL E168K N160K and HIV-2 7312A, HIV-2/HIV-1 MPER chimeric constructs. Polyclonal IgG eluted from plasma samples using tosyl activated MyOne Dynabeads coupled with RSC3 protein was tested in a neutralization assay with JR-FL WT, JR-FL D279A, DU422, CAP45.2.00 and PVO-4 pseudoviruses for evaluation of CD4BS specificity. Further, polyclonal IgG were eluted from plasma samples using tosyl activated MyOne Dynabeads coupled with MPER peptide and neutralization ability of these Abs was determined against a tier-2 pseudovirus panels (JR-FL, RHPA, CAP210 and pIndie) for evaluation of MPER specificity.

### Statistical analysis

Statistical analysis was performed using the software Graphpad prism 5.0 for determination of ID_50_ values through a dose-response curve fit with non-linear regression. Significance was calculated using t-test. P < 0.05 was considered statistically significant.

## Additional Information

**How to cite this article**: Cheedarla, N. *et al*. Broad and potent cross clade neutralizing antibodies with multiple specificities in the plasma of HIV-1 subtype C infected individuals. *Sci. Rep.* **7**, 46557; doi: 10.1038/srep46557 (2017).

**Publisher's note:** Springer Nature remains neutral with regard to jurisdictional claims in published maps and institutional affiliations.

## Supplementary Material

Supplementary Information

## Figures and Tables

**Table 1:** Clinical and Demographic profile of HIV-1 infected individuals in this study.

Samples (n = 101)*	CD4+ T cells/mm^3^		
	>350	250–350	<250
	65	21	11
**Sex**			
Men	21	10	9
Women	43	11	2
Transgender	1		
Age (Mean± SD) in years	30.5 ± 5.5	34.04 ± 7.43	42.09 ± 9.43
Median Viral Load (IQR), copies/ml	26,212(3,297–77,200)	70,200(11,570–229,500)	94,500(41,400–408,000)

A total of 101 samples were collected for the study; of these 4(*) could not be tested for CD4 count at the time of sample collection and hence not included in the above Table.

**Table 2:** Neutralization titration (ID_50_) analysis of 12 BCN plasma samples.

Sample ID	Tier-3 pseudo viruses								
	Subtype-B viruses		CRF02_AG viruses					Control	GMT
	PVO-4	TRJO4551.58	33-7	33-7	253-11	257-31	278-50	MuLV^#^	
NAB001	**2731**	**6362**	**1430**	491.7	*876*.*8*	115	93.25	<20	734
NAB004*	ND	ND	103.1	ND	ND	ND	ND	ND	NA
NAB016	**9457**	**1065**	*941*.*5*	120	116.2	76.15	138.7	<20	391
NAB033	417.1	363.7	*506*.*3*	220.8	64.07	149.3	275.1	<20	239
NAB046	69.6	**2348**	*840*.*5*	62.4	50.08	120	144.3	<20	185
NAB059	**11016**	**2062**	**8225**	427.5	*600*	49.48	**1271**	<20	**1170**
NAB062	156.7	**3029**	*929*.*3*	49.73	**1146**	53.14	50.69	<20	253
NAB063	**14915**	*927*.*2*	365.8	175.1	*718*	165.3	**1174**	<20	*741*
NAB065	*855*	268.1	361.6	296.8	136.6	75.25	100	<20	220
NAB069	**5760**	251	360	193.8	**3060**	*591*.*4*	402	<20	*688*
NAB120	47.04	114.1	*540*	262.3	**1902**	*540*	714.2	<20	343
NAB122*	95.43	234.4	ND	ND	ND	ND	ND	ND	NA

Plasma ID_50_ values are formatted as follows: italic format, ID_50_ *500*–*999* and bold format, ID_50_ ≥ **1000**. This experiment was done in duplicate on two independent occasions.

ND- Not Done; NA- Not Applicable.

*Excluded for GMT calculations. ^#^MuLV, murine leukemia virus control.

**Table 3:** Identification of CD4BS directed antibodies in BCN plasma samples.

Samples ID	Mean absorbance at 450 nm		Binding Difference between RSC3 and RSC3Δ371I/P363N proteins	P value	% of RSC3 binding antibodies in the BCN plasma samples
	RSC3 protein	RSC3D371I/P363N protein			
NAB001	0.214	0.267	−0.053	P > 0.05	ND
NAB004	0.154	0.147	0.007	P > 0.05	ND
NAB016	0.1585	0.145	0.0135	P > 0.05	ND
NAB033	**0**.**361**	**0**.**161**	**0**.**2**	**P** < **0**.**05**	**55**
NAB046	0.268	0.312	−0.044	P > 0.05	ND
NAB059	**1**.**089**	**0**.**6515**	**0**.**437**	**P** < **0**.**001**	**40**.**2**
NAB062	0.2805	0.1945	0.086	P > 0.05	ND
NAB063	**1**.**87**	**1**.**265**	**0**.**6055**	**P** < **0**.**001**	**32**.**3**
NAB065	0.084	0.153	−0.069	P > 0.05	ND
NAB069	**2**.**31**	**0**.**2085**	**2**.**102**	**P** < **0**.**001**	**91**
NAB120	0.1475	0.1615	−0.014	P > 0.05	ND
NAB122	0.237	0.1525	0.0845	P > 0.05	ND
Control (HHP)	0.075	0.079	−0.0045	P > 0.05	NA

Samples having CD4BS directed antibodies are highlighted in bold. p < 0.05 is considered significant.

N.D., Not Detectable; N.A., Not Applicable.

**Table 4:** Neutralization activity of RSC3-specific IgG eluted from four BCN plasma samples with CD4BS specificity.

Monoclonal antibody/polyclonal IgG	IC_50_ μg/ml					
	Name of pseudoviruses					
	Tier-2 subtype B		Tier-2 subtype C		Tier-3 subtype B	control virus
	JR-FL WT	JR-FL D279A	DU422	CAP45.2.00	PVO-4	MuLV
VRC01	*0*.*019*	**4**.**34**	>10	*0*.*238*	*0*.*12*	>10
b12	*0*.*02*	*0*.*10*	*0*.*08*	*0*.*31*	>10	>10
NAB069	**1**.**18**	>10	>10	**9**.**0**	**6**.**8**	>10
NAB063	**1**.**28**	>10	>10	>10	**5.2**	>10
NAB059	**9**.**1**	>10	>10	>10	>10	>10
NAB033	>10	>10	>10	>10	**9**.**0**	>10
HHP	>10	>10	>10	>10	>10	>10

IC_50_ values <0.5 μg/ml are in italics, values between 0.5–10 μg/ml are in bold format and values >10 μg/ml are normal format.

**Table 5:** Neutralization activity of BCN samples against DU156 pseudovirus with mutations in peptidoglycans targeted by the PG9/PG16 and PGT series of bNAbs.

Sample ID	ID_50_			Fold change*	
	DU156WT	N160K	N332A	N160K	N332A
NAB016	811.6	645.7	513.7	1.3	1.6
NAB033	623.8	580	310.9	1.1	2.0
NAB046	1827	7852	556.6	0.2	**3**.**3**
NAB059	1316	5500	970.7	0.2	1.4
NAB062	6400	255.8	263.6	**25.0**	**24**.**3**
NAB063	726.5	775	883.3	0.9	0.8
NAB065	468.2	609.4	184.4	0.8	2.5
NAB069	3328	1675	940	2.0	**3**.**5**
HHP	<10	<10	<10	NA	NA

*Fold change = ID_50_ of HIV-1_DU156WT_/ID_50_ of HIV-1_DU156_ mutant. Values with fold changes ≥3.0 are highlighted in bold format.

NA, Not Applicable.

**Table 6:** Neutralization activity against HIV-1_JR-FL WT_ and E168K mutant pseudoviruses.

Sample ID	ID_50_		Fold increase in the sensitivity^@^
	JR-FL WT	JR-FL E168K	E168K
NAB016	1008	5003	**4.96**
NAB033	1008	4999	**4.96**
NAB062	1232	6308	**5.12**
NAB069	922.5	12401	**13.44**

^@^The fold change calculated by ID_50_ of JR-FL E168K/ID_50_ of JR-FL WT. Increased neutralization sensitivity values >3.0 fold are highlighted in bold format.

**Table 7:** Neutralization activity of the selected BCN plasma samples against HIV-1_JR-FL E168K_ and double mutated pseudoviruses.

Sample ID	ID_50_			Fold change^#^	
	JR-FL E168K	E168K/N156K	E168K/N160K	E168K/N156K	E168K/N160K
NAB016	5003	1674	1000	2.99	**5.00**
NAB033	4999	1807	2614	2.77	1.91
NAB062	6308	1605	1584	**3**.**93**	**3**.**98**
NAB069	12401	4548	3342	2.73	**3**.**71**

^#^The fold change calculated by ID_50_ of JR-FL E168K/ID_50_ of JR-FL E168K mutant. Values ≥3.00 are highlighted in bold format.

**Table 8:** Identification of MPER specific neutralizing antibodies.

Chimera	MPER Sequence^@^	Neutralization						
		MAbs IC_50_*(μg/ml)				Plasma ID_50_ (1/dilutions)		
		2F5	4E10	Z13e1	240-D	NAB069	NAB120	NAB122
7312 A	NMYELQKLNSWDVFGNWFDLASWVKYIQYGVYIV	>10	>10	>10	>10	<20	<20	<20
C1 (clade B)	NMYELLALDKWASL**WNWFDITKWLWYIKY**GVYIV	**0**.**016**	**0**.**0017**	**0**.**08**	>10	**5**,**074**	<20	**2**,**660**
C1C (clade C)	NMYEL**LALD*****S*****W*****KN*****LWNWFDITKWLWYIKY**GVYIV	>10	**0**.**018**	**0**.**07**	>10	**12**,**960**	<20	*165*.*5*
C6	NMYELQKLNSWDVFGN**WFDIT**SWIKYIQYGVYIV	>10	**0**.**013**	>10	>10	**2**,**504**	<20	*248*.*1*
C7	NMYELQALDKWA VFGNWFDLASWVKYIQYGVYIV	**0**.**07**	>10	>10	>10	<20	<20	<20

^@^HIV-1 MPER fragment substituted in the HIV-2 7312A virus.

*Neutralization assay was performed with monoclonal antibodies 2F5, 4E10, Z13e1 as positive control and 240-D as negative control.

Plasma ID_50_ was calculated against MPER grafted HIV-2 7312A viruses. The sequence carried by the MPER of each of the constructs is indicated in the second column. Mutated residues are indicated in bold (subtype B) and *italic* (subtype C).

IC_50_ (μg/ml) formatted as **<10**; *1*–*5*; >10.

ID_50_ (dilutions) formatted as <20; *21*–*500*; >**500**.

**Table 9:** Neutralization activity of MPER-specific IgG against tier-2 pseudovirus panels.

polyclonal IgG eluted by MPER peptide	IC_50_ μg/ml				
	Name of pseudoviruses				
	Tier-2 subtype B		Tier-2 subtype C		control virus
	JR-FL	RHPA	CAP210	pIndie	MuLV
NAB069	**14**.**16**	>40	**13**.**6**	**9**.**2**	>40
NAB122	**14**.**80**	**14**.**40**	**16**.**0**	**10**	>40
HHP	>40	>40	>40	>40	>40

Neutralization titer (IC_50_) assay was performed with IgG antibody (concentrations 40 to 0.0004 μg/ml) eluted using MPER-peptide coated tosyl activated MyOne Dynabeads against four HIV-1 pseudoviruses and MuLV

IC_50_ values <20 μg/ml are shown in bold format and values >20 μg/ml are shown in normal format.

**Table 10:**
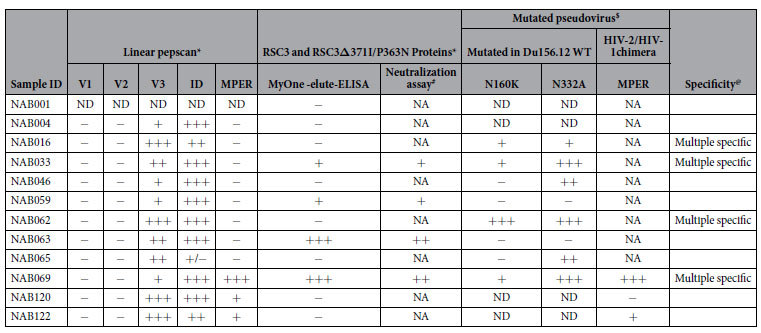
Summary of neutralization activity of the 12 BCN plasma samples.

*OD at 450 is <0.1 = −, 0.1–1.0 = +, 1.0–2.0 = ++, >2 = +++.

^$^Neutralization titer ID_50_ less than 60 = −. 61–200 = +, 201–500 = ++, >500 = +++. NA- Not Applicable, ND- Not Done.

^#^IC_50_ values <0.5 μg/ml are represented as +++, values between 0.5–5 μg/ml as ++, values >5 μg/ml as + and values >10 μg/ml as −.

^@^Neutralization analysis on HIV-1 cross clade and mutated pseudoviruses are considered for the determination of specificity.
