# Supplementary material for: Broad and potent cross clade neutralizing antibodies with multiple specificities in the plasma of HIV-1 subtype C infected individuals
Source: Sci Rep. 2017 Apr 24;7:46557. doi: 10.1038/srep46557 (PMC5402285; doi:10.1038/srep46557)
Supplement: Supplementary Information [file srep46557-s1.pdf]

Broad and potent cross clade neutralizing antibodies with multiple specificities in the plasma of HIV-1 subtype C infected individuals

Narayanaiah Cheedarla[1], Precilla Lucia K[1], Hemalatha Babu[1], Vidya Vijayan KK[1], Ashokkumar Manickam[1], Padmapriyadharshini Chendrasekeran[1], Nandagopal Kailasam[2], Jagadish Chandrabose Sundaramurthi[1], Soumya Swaminathan[1] Viswanath Buddolla[4], Kalyanaraman Vaniambadi S[3], Ramanathan V.D[1], Hanna Luke Elizabeth[1*]

[1]HIV/AIDS Division, Department of Clinical Research, National Institute for Research in Tuberculosis, Clinical Research, Chennai, India.

[2]ART Center, Kilpauk Medical College and Hospital, Chennai, India.

[3]Advanced Bioscience Laboratories Inc, Rockville, MD, USA.

[4]Department of Bionanotechnology, Gachon University, San 65, Bokjeong-Dong, Sujeong-Gu, Seongnam-Si, Gyeonggi- Do 461-701, Republic of Korea.

Key points: HIV-1, Indian subtype C, Broadly neutralizing antibodies, Multiple-epitope specificity, Tier-3 neutralization.

* Correspondence and requests for materials should be addressed to
Dr. Luke Elizabeth Hanna, (E-mail: hanna@nirt.res.in. Tel. +91-44-2836-9597)

Table S-1. Clinical and immunological demography of study samples.

Sample ID	Age (in yrs)/ Gender	Age at Diagnosis (in yrs)	CDC Clinical Stage	CD4+T Cells/mm^3	CD4/CD8 ratio	HIV-1 Subtype[#]	Viral load (viral RNA Copies/ml)	Neutralization activity in the plasma*
NAB001	37/F	32	A2	487	0.244	B/C	61,600	High
NAB002	29/F	NA	A2	494	0.403	C	15,700	Low
NAB003	28/F	NA	A2	265	0.213	C	8,740	Low
NAB004	32/M	29	A2	491	0.354	C	21,000	High
NAB005	30/F	27	A2	458	0.229	C	38,600	Low
NAB006	39/M	36	A2	384	0.235	C	103,000	Moderate
NAB007	42/M	40	A2	443	0.254	C	5,650	Moderate
NAB008	32/M	29	A2	279	0.187	C	750,000	Moderate
NAB009	34/F	NA	A2	302	0.151	C	91,100	Moderate
NAB010	45/M	43	A2	253	0.230	C	7,330	Low
NAB011	43/M	40	A2	408	0.328	C	71,300	Low
NAB012	48/F	43	A2	324	0.190	C	271,000	Moderate
NAB013	26/F	23	A2	295	0.488	C	27,300	Low
NAB014	29/F	25	A2	272	0.146	C	18,200	Low
NAB015	30/F	29	A2	403	0.349	C	253,000	Moderate
NAB016	40/M	39	A2	290	0.356	C	188,000	High
NAB017	34/F	33	A1	742	1.201	C	16,400	Low
NAB018	40/F	36	A1	1346	0.947	C	1,450	Low
NAB019	48/F	44	A3	187	0.403	C	41,400	Low
NAB020	28/F	27	A1	794	0.521	C	14,000	Moderate
NAB021	36/F	32	A1	698	0.371	C	35,000	Low
NAB022	27/M	23	A1	687	0.344	C	315,000	Low
NAB023	27/F	NA	A2	363	0.182	C	46,400	Low
NAB024	36/F	32	A1	502	0.351	C	11,600	Low
NAB025	38/F	32	A2	412	0.336	C	17,700	Moderate
NAB026	40/F	38	A1	857	0.770	C	55,700	Low
NAB027	41/M	40	A2	281	0.141	C	119,000	Low
NAB028	38/M	37	A2	319	0.287	C	13,900	Low
NAB029	40/M	39	A3	134	0.330	C	383,000	Low
NAB030	35/F	32	A2	317	0.159	C	50,600	Low
NAB031	36/F	32	A2	272	0.128	C	9,240	Moderate
NAB032	35/M	31	A2	413	0.383	C	53,700	Moderate
NAB033	33/M	28	A1	521	0.233	C	97,600	High
NAB034	29/M	NA	A2	325	0.227	C	727,000	Low
NAB035	25/F	24	A1	941	0.756	C	400	Low
NAB036	35/F	30	A2	406	0.334	C	2,530	Low
NAB037	29/F	28	A1	876	0.302	C	13,500	Low
NAB038	40/M	39	A3	130	0.081	C	408,000	Low
NAB039	31/M	30	A1	620	0.939	C	400	Low
NAB040	29/F	27	A1	674	1.272	C	<400	Low
NAB041	40/F	38	A2	256	0.239	C	180,000	Low
NAB042	46/F	45	A1	1040	1.222	C	400	Low

NAB043	33/M	31	A2	296	0.253	C	14,000	Low
NAB044	29/M	24	A1	500	0.643	C	1,020	Moderate
NAB045	43/M	39	A2	154	0.099	C	341,000	Low
NAB046	50/F	49	A2	292	0.185	C	107,000	High
NAB047	29/F	25	A2	408	0.426	C	162,000	Low
NAB048	35/F	29	A2	448	0.542	C	1,350	Low
NAB049	39/M	35	A3	175	0.348	C	555,000	Low
NAB050	45/M	41	A2	309	0.203	C	5,870	Moderate
NAB051	31/M	27	A1	766	0.328	C	456	Low
NAB052	22/M	21	A2	301	0.500	C	<400	Low
NAB053	28/F	26	A2	498	0.560	C	66,200	Low
NAB054	37/M	35	A1	1587	2.001	C	<400	Low
NAB055	29/F	25	A2	426	0.246	C	129,000	Low
NAB056	40/F	35	A1	570	0.704	C	12,700	Low
NAB057	28/F	NA	A2	372	0.375	C	750,000	Moderate
NAB058	27/F	26	A2	422	0.265	C	75,500	Low
NAB059	41/M	37	A1	507	0.659	C	380,000	High
NAB060	22/F	20	A1	832	0.509	C	2,950	Low
NAB061	32/M	NA	A1	934	0.791	C	3,860	Low
NAB062	26/F	24	A1	666	0.375	C	33,800	High
NAB063	30/M	28	A2	417	0.294	C	3,520	High
NAB064	35/F	34	A2	367	0.168	C	634,000	Moderate
NAB065	37/M	36	A2	207	0.219	C	83,400	High
NAB066	37/M	36	A1	600	0.542	C	609	Low
NAB067	24/M	NA	A3	88	0.136	C	20,800	Low
NAB068	35/F	30	A2	389	0.195	C	97,300	Low
NAB069	32/M	31	A2	210	0.335	C	94,500	High
NAB070	37/F	32	A2	370	0.426	C	9,820	Low
NAB071	31/F	27	A1	771	0.443	C	80,100	Low
NAB072	35/F	NA	A2	410	0.483	C	1,560	Low
NAB073	26/F	20	--	NA	NA	C	812	Moderate
NAB074	31F	NA	--	NA	NA	C	<400	Low
NAB075	36/F	35	A1	587	0.580	C	64,500	Low
NAB076	27/F	22	A1	545	0.708	C	4,290	Moderate
NAB077	53/M	52	A3	105	0.119	C	84,600	Low
NAB078	45/F	44	A2	326	0.460	C	711,000	Low
NAB079	44/F	43	A2	222	0.136	C	14,200	Low
NAB080	27/F	NA	A2	255	0.224	C	457,000	Low
NAB081	37/M	32	A1	823	0.708	C	55,600	Low
NAB082	33/F	28	A1	518	0.535	C	29,600	Low
NAB083	27/F	NA	A1	1172	0.474	C	292,000	Low
NAB084	33/M	30	A2	338	0.24	C	70,200	Low
NAB085	41/M	40	A1	518	0.30	C	<400	Low
NAB086	29/F	26	--	NA	NA	C	13,400	Low
NAB118	32/F	28	A2	389	NA	C	NA	Moderate
NAB119	39/M	38	A2	371	NA	C	26,212	Low
NAB120	30/F	25	A2	435	NA	C	218,816	High
NAB121	40/M	38	A3	68	NA	C	787,443	Moderate

NAB122	47/F	43	A2	477	NA	C	3,074	High
NAB123	25/F	22	A2	369	NA	C	24,593	Low
NAB124	29/F	27	A2	411	NA	C	29,444	Low
NAB125	50/M	46	A1	864	NA	C	211,836	Low
NAB126	35/M	33	A2	428	NA	C	ND	Low
NAB127	38/F	30	A1	609	NA	C	ND	Low
NAB128	29/M	28	--	NA	NA	C	ND	Low
NAB129	37/F	30	A1	642	NA	C	ND	Low
NAB130	36/M	29	A1	1034	NA	C	ND	Low
NAB131	29/M	26	A2	450	NA	C	ND	Low
NAB132	30/F	26	A2	381	NA	C	ND	Low

'A' stands for Asymptomatic, A1 CD4 ≥500 cells/ mm^3, A2 CD4 200-499 cells/ mm^3 and A3 CD4 <200 cells/ mm^3.NA-Not Available, ND-Not Done

HIV-1 subtyping has been confirmed by sequencing the *pol* and *env* genes of the all the NAB samples (Manuscript under preparation).

*Neutralization activity: Low, samples which neutralized only tier-1 pseudoviruses. Moderate, samples which neutralized tier-1 and tier-2 psedoviruses and High, samples which neutralized tier-1, tier-2 and tier-3 pseudoviruses.

Table S-2. Neutralization breadth of BCN plasma samples

**Sample ID**	**Years since diagnosis (as per records)**	**TIER-1**						**TIER-2**						**TIER-3**						**MuLV (control virus)**	**Breadth of neutralization**
		**CLADES**						**CLADES**						**CLADES**							
		**B**	**C**	**A\G**	**B-Ref**	**C-Ref**	**C-Ref**	**AG/A1**	**B**	**C**	**C**	**Indian-C (Indian Ref)**	**Indian-C**	**AG**	**AG**	**AG**	**AG**	**B-Ref**	**B-Ref**		
		**SF162.LS**	**GS015.EC 12**	**242.14**	**6535.3**	**ZM197M. PB7**	**ZM109F. PB4**	**280-5**	**TRO.11**	**CAP210.2. 00.E8**	**DU156.12**	**pINDIE**	**16936-2.21**	**33-7**	**251.18**	**253.11**	**278.50**	**PVO.4**	**TRJO455 1.58**		
NAB001	4.16	100	100	75	89	95	74	96	92	98	99	100	98	93	65	93	98	86	99	12	100%
NAB004	2.91	93	100	92	91	75	81	63	39	81	91	100	88	95	72	62	77	61	82	11	94%
NAB016	0.58	100	100	74	90	85	82	98	86	65	99	100	98	98	68	91	71	87	96	0	100%
NAB033	4.83	100	100	68	95	68	96	86	66	78	96	99	89	90	46	70	59	91	90	7	94%
NAB046	0.92	99	99	95	97	93	99	98	91	90	97	94	96	95	80	64	68	93	100	3	100%
NAB059	3.58	100	100	89	99	96	98	99	95	96	99	98	98	90	90	87	100	86	99	8	100%
NAB062	1.24	99	100	98	90	71	80	94	91	51	95	96	97	98	69	69	83	96	94	11	100%
NAB063	1.24	99	100	99	99	83	100	95	92	95	96	99	99	96	78	92	93	91	100	9	100%
NAB065	0.24	98	99	96	98	84	91	98	85	93	98	98	99	77	51	95	4	84	96	12	94%
NAB069	0.24	97	100	98	97	85	79	95	86	94	97	97	97	89	46	94	92	82	91	7	100%
NAB120	5.00	89	100	88	86	86	84	94	90	79	95	92	97	75	65	91	85	92	99	1	100%
NAB122	4.00	98	100	81	94	49	98	90	76	58	87	88	95	85	80	66	87	79	99	10	94%
HHP(-ve)	NA	<10	<10	<10	<10	<10	<10	<10	<10	<10	<10	<10	<10	<10	<10	<10	<10	<10	<10	21	NA

Neutralizing antibody screening analysis of 12 plasma samples (which were crossed tier-3 neutraliziation screening analysis, see Supporting Figure 3) against 18 pseudoviruses from different subtypes of HIV-1. The percent neutralization of the indicated pseudovirus by the indicated plasma at a screening dilution of 1/10 is shown. Plasma samples are categorized by the breadth of neutralization number of viruses neutralized at >50%; ties were broken by neutralization percentages >90% red in color, 75-90%

>90%
75-90%
51-74%
<50%

orange in color, 51-74% yellow in color and <50% no color. Pseudovirus panels tested against healthy human plasma pool sample (HHP) considered as a negative plasma control and murine leukemia virus (MuLV) used as control virus. Highlighted radiant green in color for breadth of neutralization >90%.

Supporting table S3. Neutralization activity against tier-2 pseudoviruses by MPER-specific elution.

Tier-2 Pseudovirus	**IC_{50} µg/ml from the eluted fractions**					
	NAB069-MPER	NAB069-BLANK	NAB122-MPER	NAB122-BLANK	HHP-MPER	HHP-BLANK
JR-FL	14.16	>40	14.8	>40	>40	>40
RHPA	>40	>40	14.4	>40	>40	>40
CAP210	13.6	>40	16.0	>40	>40	>40
pIndie	9.2	>40	10.0	>40	>40	>40
MuLV	>40	>40	>40	>40	>40	>40

IC_{50} values <20 µg/ml are colored yellow and values > 20 µg/ml are no color.

MuLV used as a negative virus control and HHP used as a negative plasma control.

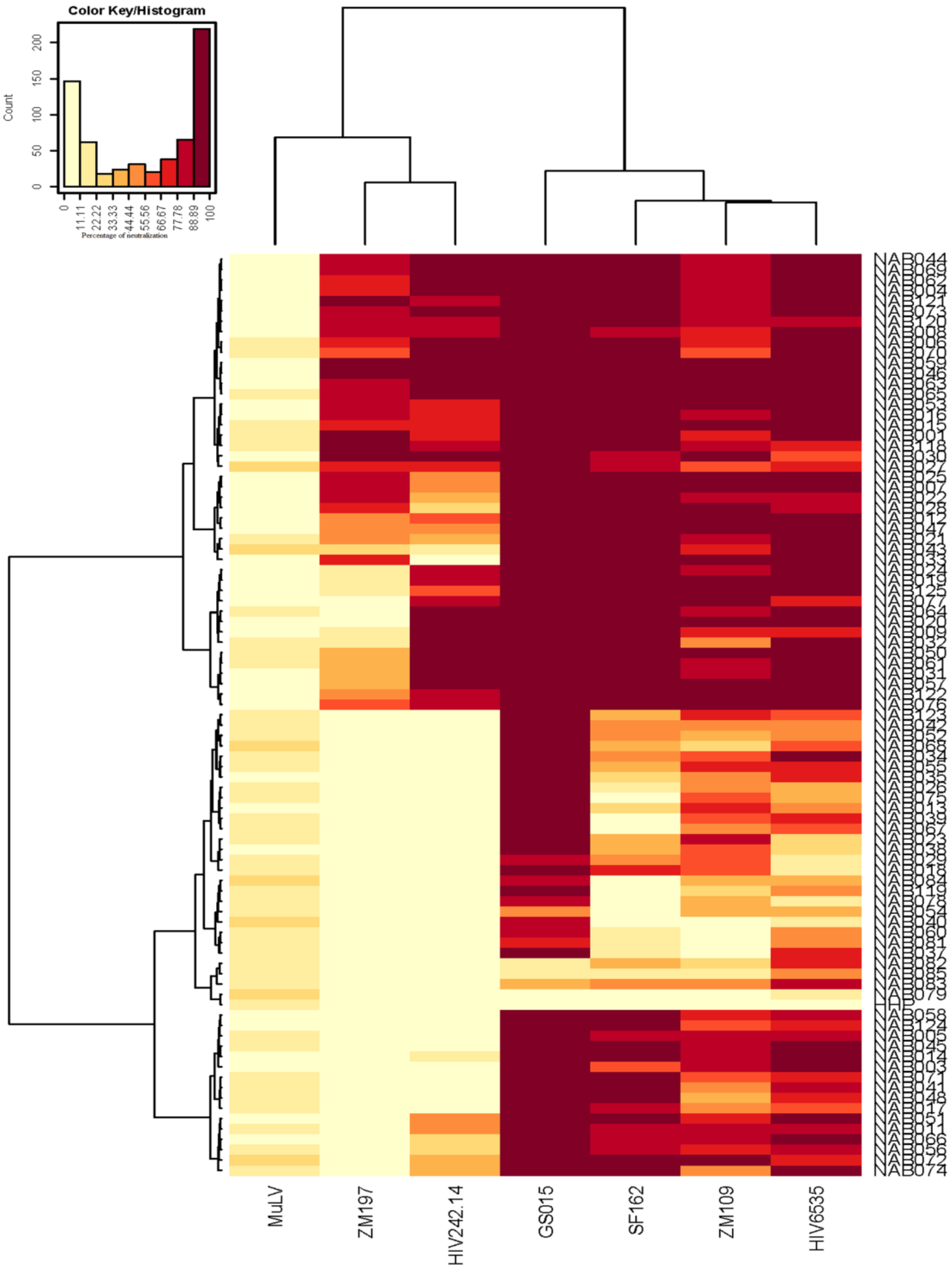

Figure S-1. Heat map developed (www.hiv.lanl.gov) from the neutralization screening analysis of study samples (n=88) and hierarchical clustering of plasma and viruses according to the neutralization percentage. The neutralization percentage of each of 88 plasma samples against 6 HIV-1 tier-1 strains and murine leukemia virus (MuLV) used as control virus. Heat map with the most potent plasmas shown in dark red and no activity in light yellow. The remaining values are binned into nine distinct colors from a palette ranging from pale yellow to dark red.

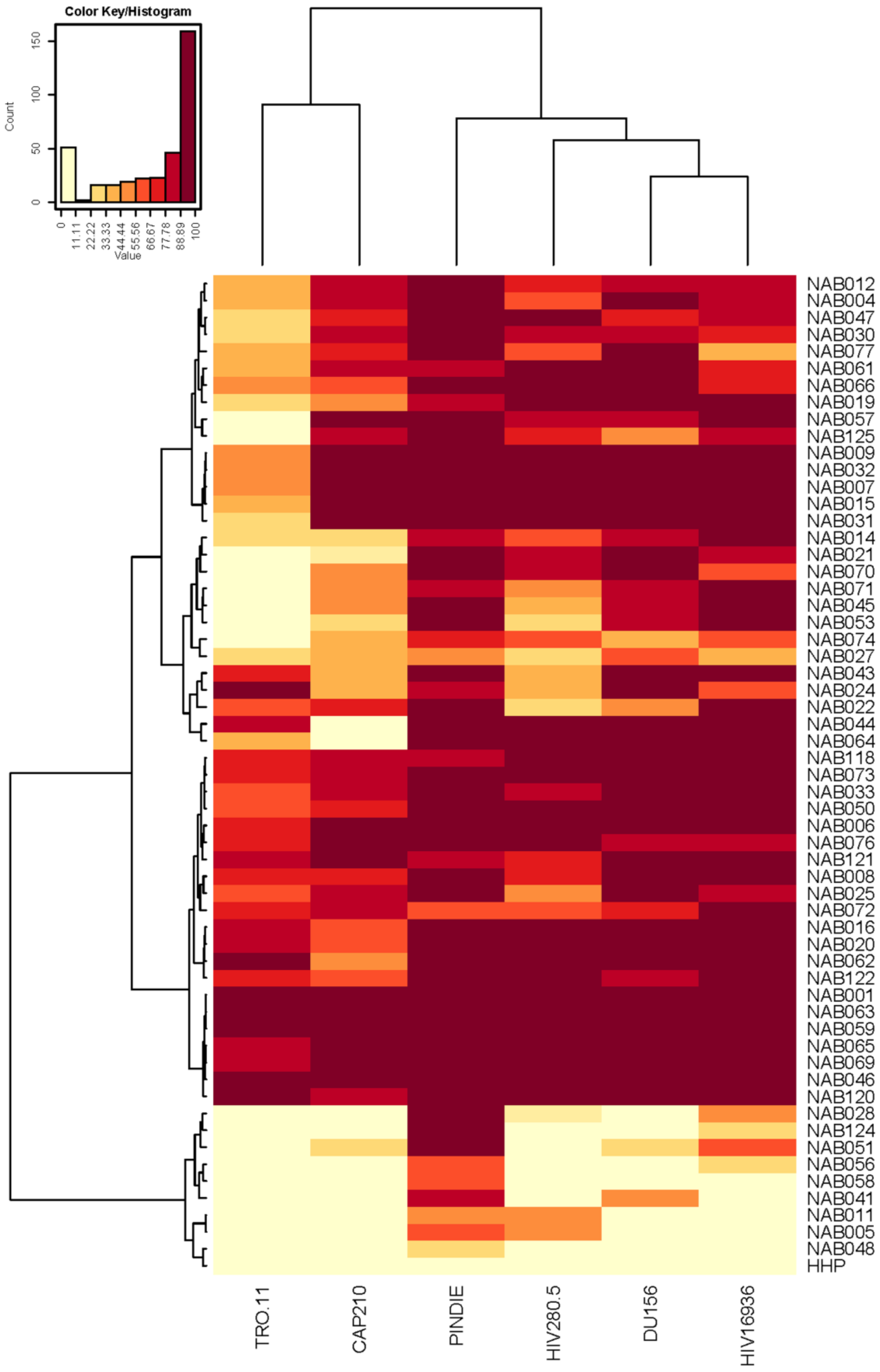

Figure S-2. Heat map developed (www.hiv.lanl.gov) from the neutralization screening analysis of tier-1 neutralization assay passed study samples (n=58) and hierarchical clustering of plasma and viruses according to the neutralization percentage. The neutralization percentage of each of 58 plasma samples against 6 HIV-1 tier-2 strains. Heat map with the most potent plasmas shown in dark red and no activity in light yellow. The remaining values are binned into nine distinct colors from a palette ranging from pale yellow to dark red.

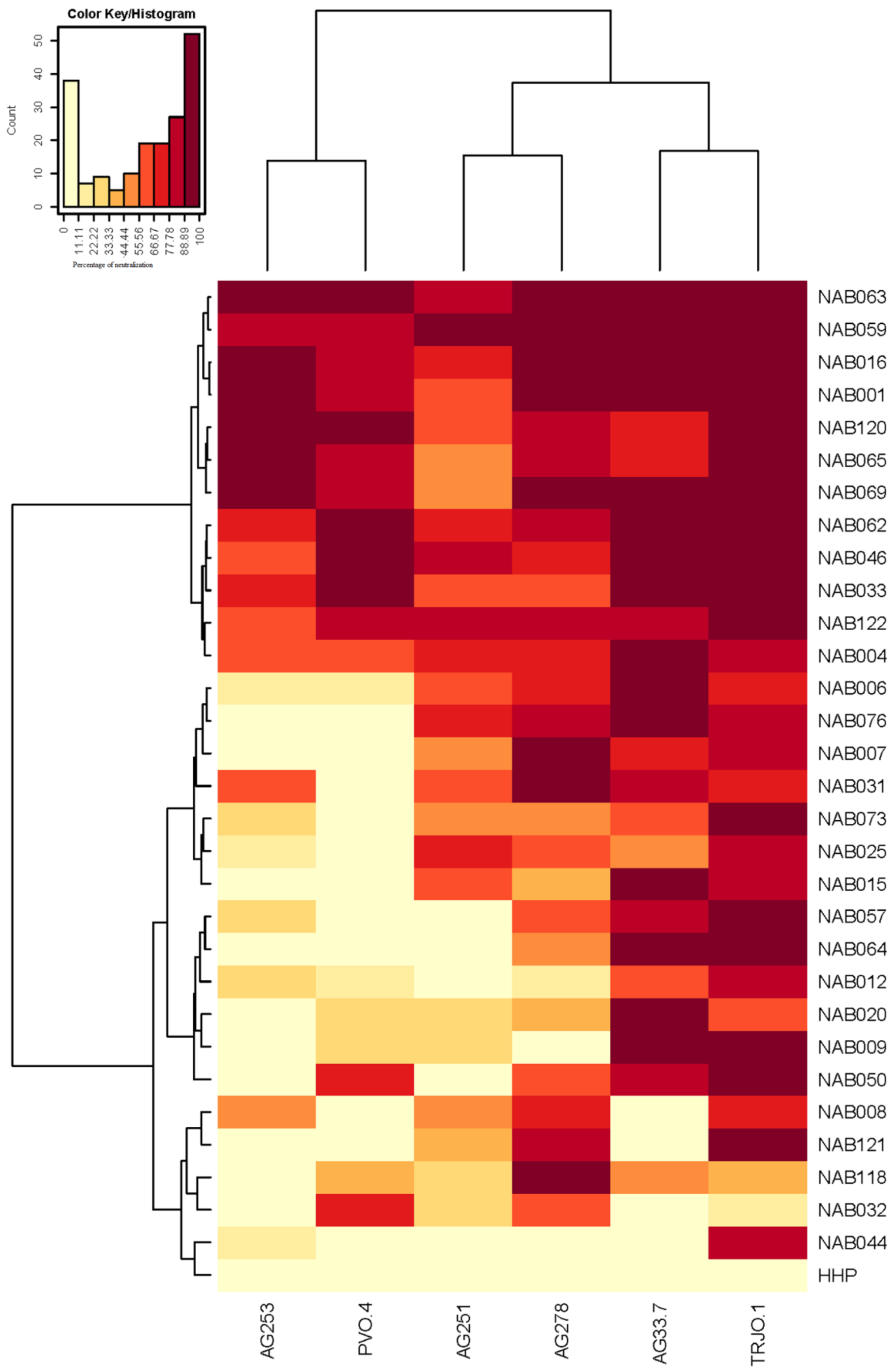

Figure S-3. Heat map developed (www.hiv.lanl.gov) from the neutralization screening analysis of tier-2 neutralization assay passed study samples (n=30) and hierarchical clustering of plasma and viruses according to the neutralization percentage. The neutralization percentage of each of 30 plasma samples against 6 HIV-1 tier-3 strains. Heat map with the

most potent plasmas shown in dark red and no activity in light yellow. The remaining values are binned into nine distinct colors from a palette ranging from pale yellow to dark red.

Top 12 plasma samples were considered as a broadly cross clade neutralizing (BCN) samples based on their neutralization breadth.

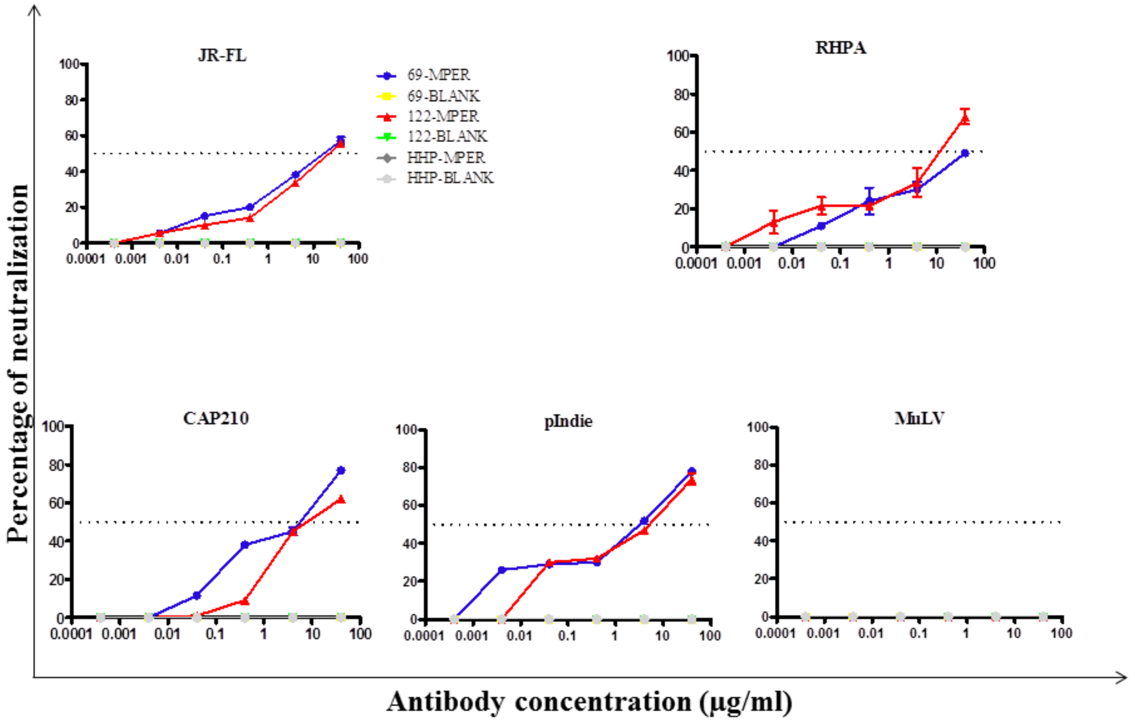

Figure S4. Neutralization titer (IC_{50}) assay performed with eluted IgG antibody concentration (from 40 to 0.0004µg/ml) of MPER peptide by tosyl activated MyOne Dynabeads against a four pseudoviruses and MuLV, murine leukemia virus control.
